# Effect of Dopamine as a Vascular Endothelial Growth Factor Antagonist on the Development of Acute Lung Injury in Sepsis Patients

**DOI:** 10.7759/cureus.64102

**Published:** 2024-07-08

**Authors:** Shivam Agarwal, Vinod K Srivastava, Zia Arshad, Pallavi Sharma, Ravi Prakash

**Affiliations:** 1 Anesthesiology and Critical Care, King George's Medical University, Lucknow, IND

**Keywords:** intensive care unit (icu), acute respiratory distress syndrome (ards), dopamine, vascular endothelial growth factor receptor (vegf), sepsis

## Abstract

Background: Sepsis is a dysregulated host immune response stemming from a systemic inflammatory response to microbial invasion, encompassing bacteria, viruses, and other pathogens. The vascular endothelial growth factor (VEGF) was initially identified for its potent induction of endothelial permeability. Studies have proposed a therapeutic role of dopamine in mitigating VEGF-induced permeability, shedding light on its potential in acute respiratory distress syndrome (ARDS) management.

Main objective: To determine the effect of dopamine as an inhibitor of VEGF and to prevent the progression of sepsis to acute lung injury (ALI) and ARDS.

Methods: A total of 154 critical care unit patients with a diagnosis of sepsis were randomized into two groups: Group I (control group) and Group II (Study group). Both received standard treatment, as per ICU protocol. In addition, the study group (Group II) received a dopamine infusion of 2 micrograms/kg/min. Baseline routine investigation, procalcitonin, and chest X-ray were done. Day one and day seven blood samples were stored for analysis of VEGF levels. Murray’s score and sequential organ failure assessment (SOFA) score (organ dysfunction) were calculated from day one to day seven.

Results: VEGF levels on day seven were significantly lower in the study group compared to the control group (p<0.05). The PaO_2_/FiO_2_ ratio at day seven was significantly increased in the study group than in the control group, indicating an improvement in oxygenation status in the study group. There was a mean ICU stay of 9.3 days in the study group versus 11.6 days in the control group (p<0.05). The SOFA score showed a significant improvement in the study group from day five onwards, indicating a therapeutic effect of dopamine on organ dysfunction in sepsis.

Conclusion: Dopamine reduces VEGF and lung injury mediated by increased endothelial permeability.

## Introduction

Sepsis is a dysregulated host response to infection, characterized by systemic inflammation, endothelial dysfunction, and organ dysfunction. Sepsis is still a diagnostic challenge, stemming from a systemic inflammatory response to microbial invasion, encompassing bacteria, viruses, and other pathogens. It ranks among the leading causes of mortality in critically ill patients as per the 2016 International Consensus Guidelines on Sepsis (version 3.0) [1]. Studies show that lysophosphatidic acid (LPA) affects a substantial proportion of sepsis patients, resulting in lung injury, ranging from 25.0% to 50.0%, and carries a mortality rate as high as 40% [2,3]. Individuals afflicted with sepsis-induced acute lung injury (S-ALI) suffer from compromised gas exchange capabilities attributed to lung inflammation and tissue impairment.

The clinical pathology of acute lung injury (ALI) includes increased vascular permeability, inflammation, oxidative stress, apoptosis, pulmonary edema, and the accumulation of activated neutrophils in lung tissue and eventually cell death [4]. Many factors and chemical mediators play a role in the pathogenesis of sepsis and its progression such as cytokines. The vascular endothelial growth factor (VEGF), also known as vascular permeability factor, was initially identified and characterized for its potent induction of endothelial permeability.

It has been shown that the normal lung contains VEGF in the alveolar space, recommending that VEGF might be an endurance factor for lung epithelial cells. VEGF levels in the epithelial coating liquid in the alveolar space are lower in patients with ARDS than in ventilated patients without ARDS [5].

Dopamine, a neurotransmitter, exerts its effects through two receptor isoforms, namely, dopamine receptors D1 and D2 (D1DR and D2DR). Activation of D2DR has also been associated with the modulation of VEGF-induced vascular permeability and tumor angiogenesis [6]. Numerous experimental and clinical investigations provide substantial evidence supporting the notion that VEGF is a key factor in the pathophysiology of acute lung injury [7,8].

In a mouse ovarian tumor model, it was previously observed that the endothelium expresses dopamine and its corresponding receptor, D2DR, thereby modulating vascular permeability and the integrity of endothelial cell barriers. Additionally, it was shown that Dopamine reduces VEGF phosphorylation [9]. Similar mechanisms are at play in murine endotoxin-induced acute lung injury (ALI), offering potential targets for enhancing outcomes in this preclinical model and potentially in humans. Sepsis stands out as the primary cause of ALI in human patients [10].

The study conducted in 2008 proposes a novel therapeutic role for dopamine, shedding light on its potential in a new therapeutic context. Furthermore, this report elucidates the molecular mechanism underlying dopamine's impact on VPF/VEGF-induced permeability [11].

In addition to dopamine, oxidative stress and hypoxia contribute to the expression of VEGF. This suggests the potential for a positive feedback loop among these factors, amplifying VEGF expression. Consequently, VEGF binding to its receptors may contribute to heightened vascular permeability and increased hypoxemia, characteristics observed in the early stages of ARDS. This study marks the first association of a common variant in the FTL1 gene with susceptibility to sepsis-induced ARDS [12]. Thus, after determining the role of dopamine in sepsis-induced pathophysiology, we have planned a trial of low-dose dopamine infusion in sepsis patients. This study aimed to determine the effect of dopamine as a VEGF antagonist on the development of acute lung injury in sepsis.

The main objective here is to determine the effect of dopamine as an inhibitor of VEGF and to prevent the progression of sepsis to ALI and ARDS. The secondary objectives are to establish the relationship between VEGF level in severity of lung injury and to evaluate the effect of dopamine on the length of ICU stay.

## Materials and methods

Methodology

This randomized control study was conducted in a tertiary-level intensive care unit (ICU) over a period of one year, after getting ethical approval (Institutional Ethics Committee, King George's Medical University, Lucknow (Ref. code XIV-PGTSC-IIA/P9) and CTRI registration (CTRI/2023/05/052798).

We included adult patients admitted to the ICU with the diagnosis of sepsis. The exclusion criteria include patients having ARDS, head injury, preexisting lung, and cardiovascular disease. ICU mortality within 48 hours or loss to follow-up was also excluded. We monitor the VEGF levels and the development of ALI on the basis of Murray’s score and PaO_2_/FiO_2_ ratio.

**Figure 1:**
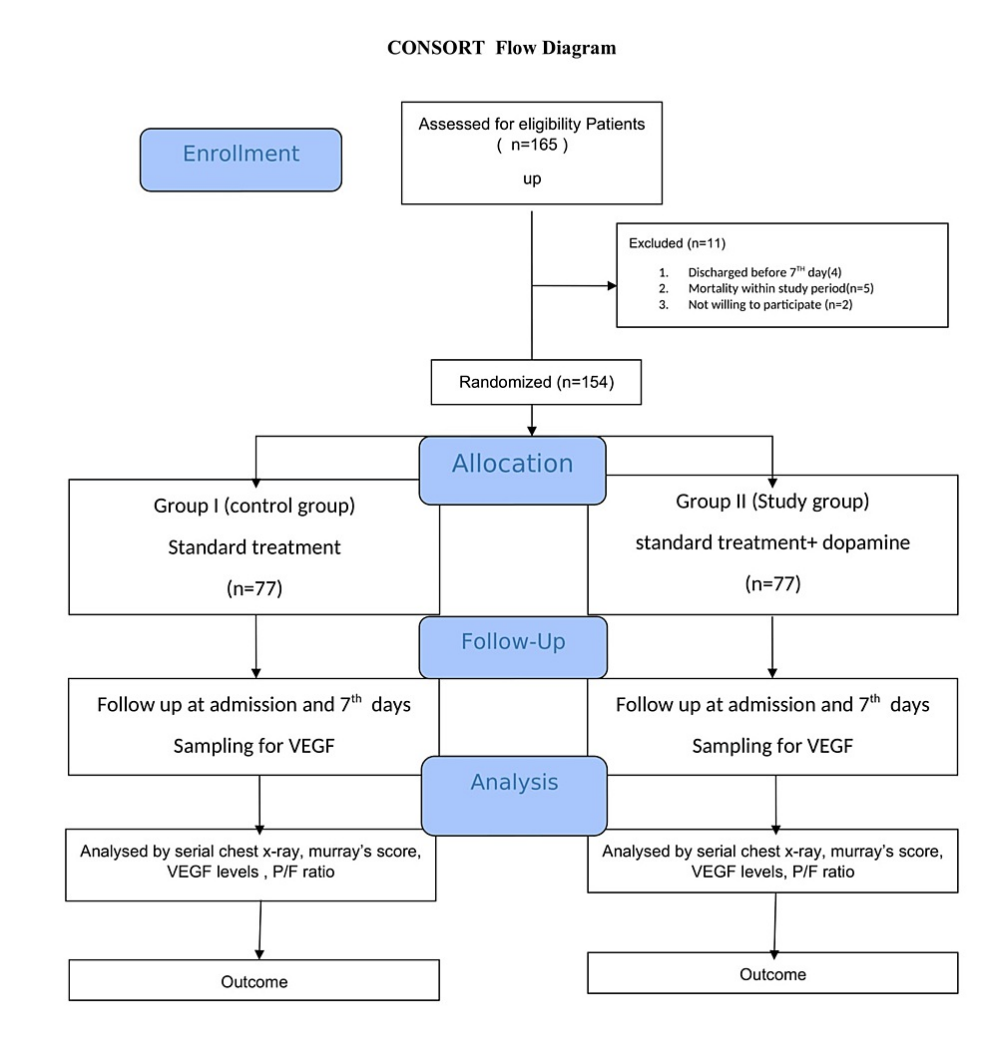
CONSORT diagram CONSORT: Consolidated Standards of Reporting Trials

The sample size was calculated based on a previous study reporting that the incidence of ALI/ARDS was 11.4% in India [13] and 95% level of confidence, and the error rate, usually set at the 0.07 level is, 4. A total of 154 patients were included in this study. The sample size was calculated by using the formula, n=Z2 P(1-P) / d2, where n=sample size; Z statistic for a level of confidence and for the level of confidence of 95%, which is conventional, with Z value of 1.96; P=expected prevalence or proportion (in proportion of one; and (if 11.4%, P = 0.11), d=precision (in proportion of one; if 7%, d=0.07) [13]. The diagram is presented in Figure 1.

A total of 154 patients were randomly allocated in two groups. Both groups received standard treatment. In the study group besides standard treatment, dopamine infusion was started at a rate of 2 micrograms/kg/min, while a placebo/saline infusion was given for patients in the control group. Dopamine infusion was stopped in patients who developed arrhythmia.

Apart from demographic data, the baseline vitals (blood pressure, heart rate, oxygen saturation at the time of admission) and parameters recorded at the time of admission include arterial blood gas (ABG) analysis, pro-calcitonin (PCT), and chest X-ray (CXR). Acute Physiology and Chronic Health Evaluation II (APACHE II) and Sequential Organ Failure Assessment (SOFA) scores were assessed after admission, and the severity and daily progress of the disease was assessed by SOFA scoring, CXR findings, PaO_2_/FiO_2_, and Murray’s acute lung injury score. 2D-ECHO was done on the day of admission. Levels of VEGF were done at the time of admission and on the seventh day. Samples were collected and sent for analysis by the personnel working in the ICU so that neither the patient nor the investigator was aware of which treatment the patient had received.

Statistical analysis was performed using Statistical Product and Service Solutions (SPSS, version 21.0; IBM SPSS Statistics for Windows, Armonk, NY). The data were expressed as mean (standard deviation) for continuous variables and numbers with percentage (%) for categorical variables. The chi-square test was employed to compare categorical variables, while the independent t-test was utilized to compare discrete variables between groups. The sensitivity, specificity, PPV, NPV, and ROC curves were used to assess the overall diagnosis of lung injury. Regression analysis was used for the estimation of relationships between a dependent variable and one or more independent variables. The p-value of 0.05 is to be considered significant.

## Results

Statistical analysis was carried out by using SPSS (version 21.0), and the following observations were recorded.

**Figure 2:**
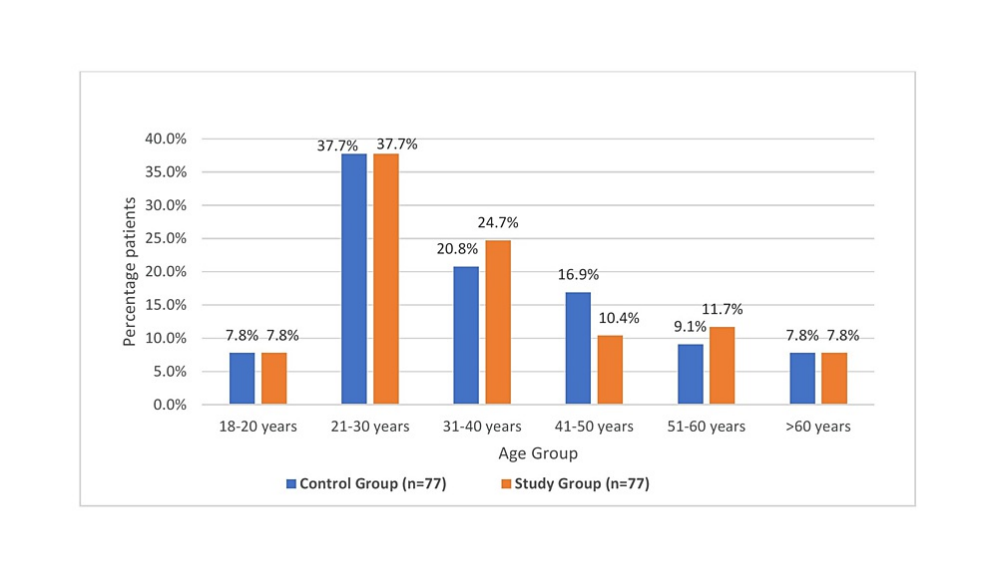
Age in both groups n: number of patients in each group

Both the groups are comparable demographically, as shown in Table 1 and Figure 2. ABG parameters did not differ significantly between the control and study groups, indicating comparable respiratory and metabolic status among the patients in both groups (Table 2). Baseline hemodynamic parameters were also comparable in both groups (Table 3).

**Table 1:** Gender distribution

Gender	Male	42 (54.5)	41 (53.2)	p-value=0.871
	Female	35 (45.5)	36 (46.8)	

**Table 2:** Baseline ABG parameters in both groups ABG: arterial blood gas

Baseline ABG Parameters	Control Group (n=77) (mean±SD)	Study Group (n=77) (mean±SD)	p-value
PO_2_ (mmHg)	128.2±39.9	137.3±53.5	0.235
PCO_2_ (mmHg)	34.9±5.3	35.7±5.7	0.348
Lactate	1.6±0.79	1.5±0.74	0.470
FiO_2_ (%)	39.9±12.0	39.9±10.3	0.989
PO_2_/FiO_2_	331.1±77.2	347.6±98.8	0.253

**Table 3:** Baseline parameters

Hemodynamics	Control Group (n=77) (mean±SD)	Study Group (n=77) (mean±SD)	p-value
Heart rate/minute	90.6±10.7	92.5±13.0	0.331
Respiratory rate/minute	18.8±2.3	19.0±2.7	0.659
SPO_2_	99.0±0.7	98.8±0.8	0.410
MAP (mean arterial pressure) mmHg	87.7±8.8	86.3±8.7	0.309
Procalcitonin	8.7±8.6	12.8±17.1	0.065

The mean Murray's score on day one in the study group was slightly lower, but this difference was not statistically significant. On day two, the difference in mean Murray's scores was found to be statistically significant. However, from days three to seven, there were no statistically significant differences observed between the two groups (Table 4).

**Table 4:** Murray’s score in two groups

Murray's Score	Control Group (n=77) (mean±SD)	Study Group (n=77) (mean±SD)	p-value
Day-1	0.38±0.3	0.28±0.2	0.056
Day-2	0.38±0.3	0.27±0.2	0.035
Day-3	0.40±0.4	0.32±0.4	0.210
Day-4	0.40±0.4	0.32±0.4	0.292
Day-5	0.44±0.5	0.34±0.4	0.236
Day-6	0.48±0.5	0.39±0.4	0.267
Day-7	0.49±0.5	0.40±0.5	0.315

The baseline mean PaO_2_/FiO_2_ ratio was comparable in both groups. On subsequent days, significant differences were observed. On day two, the differences in mean ratios were statistically significant. From day three onwards, the study group showed higher PaO_2_/FiO_2_ ratios compared to the control group, which was consistently higher in the study group till day seven and continuously decreased in the control group (Table 5).

**Table 5:** Comparison of the PO2/FiO2 ratio between the two groups

PO_2_/FiO_2_ ratio	Control Group (n=77) (mean±SD)	Study Group (n=77) (mean±SD)	p-value
Day-1	326.9±76	347.6±98	0.150
Day-2	302.1±48	324.0±45	0.005
Day-3	309.7±66	325.7±49	0.090
Day-4	297.8±58	315.9±55	0.051
Day-5	291.9±67	320.5±60	0.007
Day-6	286.2±70	326.3±73	0.001
Day-7	284.4±83	330.5±88	0.001

On day one, the mean VEGF level in the control group was 169.3 ± 65.5 pg/mL, while in the study group, it was slightly lower; however, this difference was not statistically significant. On day seven, a notable disparity was observed between the two groups. The mean VEGF level in the control group substantially increased, whereas in the study group, it decreased. This difference was statistically significant. In the control group, the mean difference between VEGF levels on day one and day seven was -27.48, indicating a decrease in VEGF levels from day one to day seven. This difference was statistically significant, suggesting that VEGF levels significantly decreased from day one to day seven in the control group. Conversely, in the study group, the mean difference in VEGF levels between day one and day seven was not statistically significant (Tables 6-8).

**Table 6:** Distribution of patients based on their VEGF levels in both groups VEGF: vascular endothelial growth factor

VEGF Levels	Control Group (n=77) (mean±SD)	Study Group (n=77) (mean±SD)	p-value
Day-1	169.3±65.5	158.0±65.5	0.287
Day-7	196.8±77.3	147.8±74.6	<0.001

**Table 7:** Paired differences in VEGF levels in the control group VEGF: vascular endothelial growth factor

Paired Samples Test								
Control Group (n=77)	Paired Differences					t	df	Sig. (2-tailed)
	Mean	Std. Deviation	Std. Error Mean	95% Confidence Interval of the Difference				
				Lower	Upper			
VEGF Levels day 1 vs VEGF Levels day 7	-27.48	73.62	8.39	-44.19	-10.76	-0.3.2	76	0.002

**Table 8:** Paired differences in VEGF levels in the study group VEGF: vascular endothelial growth factor

Paired Samples Test								
Study Group (n=77)	Paired Differences					t	df	Sig. (2-tailed)
	Mean	Std. Deviation	Std. Error Mean	95% Confidence Interval of the Difference				
				Lower	Upper			
VEGF Levels day 1 vs VEGF Levels day 7	10.18	75.90	8.65	-7.04	27.40	1.17	76	0.243

Significantly more patients in the study group had an ICU stay of 10 days or less compared to the control group, and the difference is statistically significant. Conversely, a higher proportion of patients in the control group had an ICU stay exceeding 10 days compared to the study group. Additionally, the mean length of ICU stay was significantly shorter in the study group compared to that in the control group (Table 9).

**Table 9:** Distribution of patients based on their ICU stay in both groups

Length of ICU Stay (day)	Control Group (n=77)	Study Group (n=77)	p-value
≤10 day	26 (33.8%)	61 (79.2%)	<0.001
>10 day	51 (66.2%)	16 (20.8%)	
Mean±SD	11.6±2.5	9.3±1.9	<0.001

The difference in mean APACHE II scores between the two groups was comparable (Table 10).

**Table 10:** APACHE II score in both groups

APACHE II	Control Group (n=77) (mean±SD)	Study Group (n=77) (mean±SD)	p-value
Score	15.7±5.4	14.6±6.1	0.236

**Figure 3:**
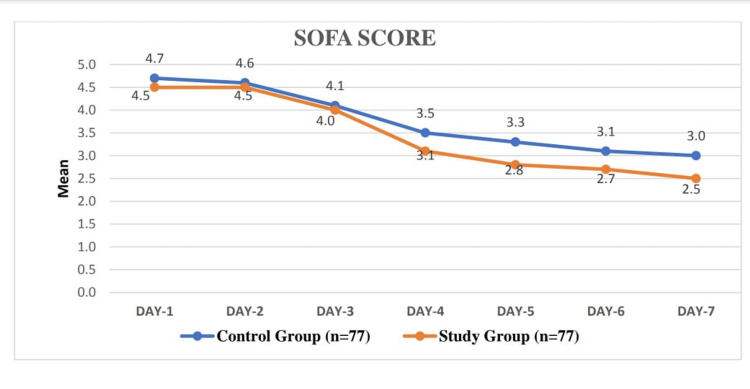
SOFA score in two groups SOFA: sequential organ failure assessment

Table 11 shows the mean SOFA scores on days one through seven in the study and control groups. The mean SOFA scores for the corresponding days were slightly lower. The p-values associated with the comparison of SOFA scores between the two groups varied across the days. While there were no statistically significant differences on days one, two, three, and four, significant differences emerged on days five, six, and seven (Table 11, Figure 3).

**Table 11:** Comparison of SOFA score between the two groups SOFA: sequential organ failure assessment

SOFA Score	Control Group (n=77) (mean±SD)	Study Group (n=77) (mean±SD)	p-value
Day-1	4.7±1.3	4.5±1.9	0.451
Day-2	4.6±1.4	4.5±1.8	0.504
Day-3	4.1±1.7	4.0±1.7	0.563
Day-4	3.5±1.9	3.1±2.0	0.076
Day-5	3.3±2.0	2.8±2.0	0.026
Day-6	3.1±1.9	2.7±2.0	0.035
Day-7	3.0±2.0	2.5±2.0	0.050

In the control group, the mean paired differences between SOFA score 1 (baseline) and subsequent days (SOFA score 2 to SOFA score 7) were statistically significant for all comparisons. Notably, the mean differences increased progressively from day two to day seven, indicating a worsening trend in organ dysfunction. Similarly, in the study group, significant differences were observed between SOFA scores on day one and subsequent days, with increasing mean differences over time (Tables 12-13). The p value<0.001 is considered significant.

**Table 12:** Paired differences in the SOFA score of the control group SOFA: sequential organ failure assessment

Paired Samples Test								
Control Group (n=77)	Paired Differences					t	df	Sig. (2-tailed)
	Mean	Std. Deviation	Std. Error Mean	95% Confidence Interval of the Difference				
				Lower	Upper			
SOFA_SCORE_1 vs SOFA_SCORE_2	0.06494	0.24803	0.02827	0.00864	0.12123	2.297	76	0.024
SOFA_SCORE_1 vs SOFA_SCORE_3	0.55844	1.11796	0.12740	0.30470	0.81219	4.383	76	0.000
SOFA_SCORE_1 vs SOFA_SCORE_4	1.10390	1.31374	0.14971	0.80571	1.40208	7.373	76	0.000
SOFA_SCORE_1 vs SOFA_SCORE_5	1.32468	1.41832	0.16163	1.00276	1.64659	8.196	76	0.000
SOFA_SCORE_1 vs SOFA_SCORE_6	1.48052	1.40122	0.15968	1.16248	1.79856	9.272	76	0.000
SOFA_SCORE_1 vs SOFA_SCORE_7	1.70130	1.55652	0.17738	1.34801	2.05459	9.591	76	0.000

**Table 13:** Paired differences in the SOFA score of the study group SOFA: sequential organ failure assessment

Paired Samples Test								
Study Group (n=77)	Paired Differences					t	df	Sig. (2-tailed)
	Mean	Std. Deviation	Std. Error Mean	95% Confidence Interval of the Difference				
				Lower	Upper			
SOFA_SCORE_1 vs SOFA_SCORE_2	0.03896	0.41172	0.04692	0.05449	0.13241	0.830	76	0.409
SOFA_SCORE_1 vs SOFA_SCORE_3	0.51948	0.82095	0.09356	0.33315	0.70581	5.553	76	0.000
SOFA_SCORE_1 vs SOFA_SCORE_4	1.45455	1.31335	0.14967	1.15645	1.75264	9.718	76	0.000
SOFA_SCORE_1 vs SOFA_SCORE_5	1.79221	1.50733	0.17178	1.45009	2.13433	10.433	76	0.000
SOFA_SCORE_1 vs SOFA_SCORE_6	1.90909	1.68727	0.19228	1.52613	2.29205	9.929	76	0.000
SOFA_SCORE_1 vs SOFA_SCORE_7	2.09091	1.87912	0.21415	1.66440	2.51742	9.764	76	0.000

## Discussion

Sepsis remains a significant global health challenge, with high morbidity and mortality rates despite advances in critical care medicine [14]. Sepsis is characterized by systemic inflammation, endothelial dysfunction, and organ dysfunction. Despite advances in supportive care, sepsis remains a leading cause of mortality worldwide, with a mortality rate ranging from 20% to 30%. ALI and ARDS are frequent complications of sepsis, contributing to respiratory failure and death in critically ill patients [15].

The pathophysiology of S-ALI involves complex interactions between inflammatory mediators, endothelial dysfunction, and impaired alveolar fluid clearance [16]. VEGF, a potent angiogenic factor, plays a central role in vascular permeability regulation and endothelial barrier integrity. In sepsis, dysregulated VEGF signaling contributes to increased microvascular permeability, leading to pulmonary edema and ALI development [17]. Additionally, vascular endothelial growth factor is found to be an important determinant of sepsis morbidity and mortality [18].

Dopamine, a neurotransmitter, acts through two receptor isoforms, namely, D1DR and D2DR. Research indicates that D1DR enhances transepithelial fluid flux by facilitating the movement of Na-K ATPase to the basolateral membrane of type II alveolar epithelial cells. Similarly, activation of D2DR stimulates the expression of Na-K ATPase genes. However, dopamine's effects on pulmonary vascular barrier properties extend beyond sodium transport regulation. D2DR activation is also associated with the modulation of VEGF-induced vascular permeability and tumor angiogenesis. Recent studies have implicated dopamine as a potential modulator of VEGF signaling pathways, suggesting its therapeutic potential in ALI attenuation. By antagonizing VEGF-mediated endothelial dysfunction, dopamine may mitigate ALI progression and improve patient outcomes in sepsis [19].

We aimed to evaluate the effect of dopamine as a VEGF antagonist on the development of ALI in septic patients. The findings of this study provide valuable insights into potential preventive interventions for ALI in sepsis.

In our study of 154 patients, evenly divided between control and study groups, no significant differences were found in age or gender composition between the two groups (p>0.05). Overall, both groups exhibited comparable demographic characteristics.

The blood test results, including total leukocyte count and platelet count, showed no significant differences between the control and study groups (p>0.05); therefore, baseline haematological parameters were similar, minimizing their influence on the study outcomes.

The baseline ABG parameters, including PO_2 _(p=0.235), PCO_2 _(p=0.348), lactate levels (p=0.470), and derived parameters such as PO_2_/FiO_2_ (p=0.253) ratio, exhibited no significant differences between the two groups. These findings indicate that the respiratory and metabolic status of patients were comparable at baseline.

Baseline vitals such as heart rate (HR) (p=0.331) and respiratory rate (RR) were comparable between the groups (p=0.659). The mean arterial pressure (MAP) had a slightly higher mean value in the control group, though this difference was not statistically significant (p=0.309). Oxygen saturation (SPO_2_) levels were also similar between the two groups (p=0.410). The mean procalcitonin levels were higher in the study group (12.8±17.1 ng/mL) compared to the control group (8.7±8.6 ng/mL), although this difference was not statistically significant (p=0.065).

On day one, the mean VEGF level was 169.3±65.5 in the control group and 158.0±65.5 in the study group, with no significant difference observed between the groups (p=0.287). However, by day seven, a substantial contrast emerged, with the control group showing a mean VEGF level of 196.8±77.3 compared to 147.8±74.6 in the study group, signifying a significant decrease in VEGF expression in the study group (p<0.001). This suggests a potential impact of the intervention or treatment on VEGF regulation, which could have implications for angiogenesis, tissue repair, and overall patient recovery. We found a significant reduction in VEGF levels on day seven in the study group compared to the control group where it rose from the baseline value. This indicates that dopamine administration may attenuate VEGF production, which could potentially mitigate endothelial dysfunction and ALI progression.

This finding has also been supported by some previous studies such as Sarkar et al. who found that D2DRs, upon activation, inhibit the proangiogenic actions of VEGF-A, also known as vascular permeability factor [20].

Ferrero et al. also favours our study and found that D2-ag inhibits VEGF secretion at the post-transcriptional level, suggesting that D2-ag treatment should be combined with therapies that inhibit VEGF transcription [21].

Basu et al. reported that, at non-toxic levels, the neurotransmitter dopamine strongly and selectively inhibited the vascular permeabilizing and angiogenic activities of VPF/VEGF. Dopamine acted through D2DRs to induce endocytosis of VEGF receptor 2. To assess the acute lung injury in enrolled patients, we used Murray’s acute lung injury score’, which comprises four components - PaO_2_/FiO_2_ ratios, consolidation on chest radiograph, positive end-expiratory pressure (PEEP), and respiratory compliance [22].

On day one, the mean Murray's score was 0.38±0.3 in the control group and 0.28±0.2 in the study group, with no statistical significance (p=0.056). From day two to day seven, there were no statistically significant differences observed between the two groups, with p-values ranging from 0.210 to 0.315.

Thus, according to Murray’s score, lung injury progressed in both groups of patients, but by day seven, the severity of lung injury was found to be less in the study group (0.40±0.5) than in the control group (0.49±0.5), although this difference was not significant and Murray’s score was comparable in both the groups (p=0.315).

Therefore, to assess the level of lung injury and to co-relate it with the VEGF levels, we compared day one and day seven PaO_2_/FiO_2_ ratios of the study and control groups with the corresponding VEGF levels.

We found that on day one, the mean PaO_2_/FiO_2_ ratio was 326.9±76 in the control group and 347.6±98 in the study group, with a p-value of 0.150. On subsequent days, significant differences were observed. From day three onwards, the study group showed higher PaO_2_/FiO_2_ ratios compared to the control group, which were consistently higher in the study group than the control group till day seven. On day seven, the mean PaO_2_/FiO_2_ ratio was 330.5±88, which was significantly higher than the day seven values, 284.4±83 in the control group, with p-values of 0.001 and 0.001, on day sixth and seventh, respectively. This finding was consistent with the fact that there significant reduction in VEGF levels on day seven (147.8±74.6) in the study group compared to the control group (196.8±77.3), from their baseline value. This indicates that, although lung injury developed in both groups, as per Murray’s score, the PaO_2_/FiO_2_ ratio and thus oxygenation status were found to be improved in the study group. However, this trend suggests a potential benefit of dopamine as a VEGF antagonist in improving pulmonary function and reducing ALI severity.

This improvement in lung function was consistent with the findings in a study by Vohra et al. in which he demonstrated that dopamine prevents pulmonary edema through the VEGF-VEGFR2 axis in a murine model of acute lung injury [7]. They also concluded that dopamine acts through D2DR to inhibit pulmonary edema-associated vascular permeability, which is mediated through VEGF-VEGFR2 signalling and conveys protective effects in an ALI model.

Another study by Meng et al. also demonstrated that D1DR agonists inhibit acute lung injury via modulating inflammatory responses in macrophages and barrier function in airway epithelial cells and found that SKF is a selective agonist for D1-like receptors and was demonstrated to inhibit excessive inflammatory responses and oxidative stress in THP-1 cell-derived macrophages and Beas-2B cells, as well as improve airway epithelial barrier dysfunction induced by LPS stimulation [23].

The mean length of ICU stay was notably shorter in the study group, with a mean of 9.3±1.9 days compared to 11.6±2.5 days in the control group, reflecting a statistically significant difference (p<0.001). The study group exhibited a significantly shorter ICU stay compared to the control group. This finding suggests that dopamine as a VEGF antagonist may contribute to early recovery and reduced ICU utilization in septic patients.

In the control group, the mean APACHE II score was 15.7±5.4, while in the study group, it was slightly lower at 14.6±6.1. The APACHE II score did not differ significantly between the groups (p>0.05).

We also studied the effect of dopamine on the overall survival of ICU patients by measuring the SOFA score. It consists of six components - PaO_2_/FiO_2_ ratios, platelet count, bilirubin levels, hemodynamic status, Glasgow coma score, and serum creatinine levels.

Initially, on day one, the mean SOFA scores were comparable between the control group (4.7±1.3) and the study group (4.5±1.9), with a non-significant p-value of 0.451. However, as the days progressed, differences emerged. By day five and onwards, the study group exhibited lower mean SOFA scores compared to the control group, indicating less severe organ dysfunction. These differences became statistically significant on days five, six, and seven, with p-values of 0.026, 0.035, and 0.050, respectively, suggesting a potential improvement in organ function in the study group over time. This finding correlates with a study done by Moore et al. in which he studied the anti-inflammatory effects of peripheral dopamine and found that dopamine is also synthesized in a number of peripheral organs, as well as in several types of cells, and has organ-specific functions and is involved in the regulation of the immune response and inflammatory reaction [24].

Limitations of the study

The study included a relatively small sample size, which may limit the generalizability of the findings to a broader population. A larger sample size would provide more robust results and enhance the external validity of the study. The study was conducted at a single center, which limits the diversity of patient populations and healthcare practices. Multi-centric studies would provide a more comprehensive understanding of the intervention's effectiveness across different settings.

## Conclusions

Dopamine, which acts as a VEGF antagonist, was found to decrease the level of VEGF in patients with sepsis. Dopamine infusion in low dosage improves lung function with respect to a better PaO_2_/FiO_2_ ratio. It was found that there were improved SOFA scores as well as improved disease severity. The study also reveals that the length of ICU stay is shorter in patients who received low-dose dopamine, the VEGF antagonist infusion during their treatment.
